# Clinical Course and Treatment Outcomes of Vulvar Condylomas in Immunocompromised Women: A Retrospective Cohort Study

**DOI:** 10.1007/s12026-026-09843-6

**Published:** 2026-09-12

**Authors:** Fernanda Villar Fonseca, Camilly Eduarda Kmita, Carlos Afonso Maestri, Isabela Ceschin Maestri, Renato Nisihara

**Affiliations:** 1 Department of Medicine, Universidade Positivo, R. Prof. Pedro Viriato Parigot de Souza, 5300, Curitiba, 81290-000 Brazil; 2 Department of Cervical Pathology and Colposcopy, Hospital Erasto Gaerner, Curitiba, Brazil; 3 Federal University of Paraná, Curitiba, Brazil

**Keywords:** Human papillomavirus, Vulvar condyloma, Immunosuppression

## Abstract

Human papillomavirus (HPV) infection is highly prevalent worldwide and is associated with a broad spectrum of anogenital diseases, including vulvar condylomas and malignancies. Immunosuppression plays a key role in HPV persistence, recurrence, and progression; however, data on the clinical course of vulvar condylomas in immunocompromised women remain limited. This retrospective cohort study evaluated clinical progression, treatment response, recurrence, and neoplastic outcomes in women diagnosed with vulvar condyloma between January 2008 and January 2021 at a tertiary referral center in Brazil. Immunosuppressed patients, including women living with HIV, those receiving immunosuppressive therapy for autoimmune diseases, and patients undergoing chemotherapy, were compared with an age-matched immunocompetent group. Among 294 women included, 41 (13.9%) were immunosuppressed. Lesion extent was significantly greater in immunosuppressed patients, although lesion number did not differ between groups. Clinical remission after first-line treatment was significantly lower among immunosuppressed women (21.9%vs55.0%; *p* < 0.0001; OR = 4.5) with a higher need for multiple treatment sessions. Similarly, the complete remission rate at the end of follow-up was higher for the immunocompetent patients (75.9% vs. 48.8%; *p* < 0.0001; OR = 3.3). Immunosuppressed women had a 2.3-fold increased risk of recurrence and a higher risk of progression to vulvar intraepithelial neoplasia (OR = 6.7) and invasive disease (OR = 8.5). In the HIV patients, recurrence was not associated with CD4T-lymphocyte count or HIV viral load. These findings indicate that immunosuppressed women with vulvar condylomas experience more extensive disease, poorer treatment response, higher recurrence rates, and an increased risk of neoplastic progression, highlighting the importance of closer surveillance and individualized management in this high-risk population.

## Introduction

Human papillomavirus (HPV) is the most prevalent sexually transmitted infection worldwide and a leading etiologic agent of cervical cancer, as well as other anogenital and oropharyngeal malignancies. Immunosuppression represents one of the main factors favoring HPV persistence and progression to high-grade lesions and cancer [1]. Immunosuppressed individuals present a significantly increased risk of developing aggressive and recurrent HPV-related diseases, particularly when infected with oncogenic viral types [2]. Persistent HPV infection in this population is closely associated with impaired innate and adaptive immune responses [2]. Among people living with HIV, reduced CD4⁺ T-lymphocyte counts and persistent viral replication further compromise immune control of HPV infection. HIV co-infection may increase the risk of progression to high-grade intraepithelial neoplasia or cervical cancer by up to fourfold, and even individuals with undetectable viral load remain susceptible to recurrence due to residual immune dysfunction [3].

Vulvar condylomas are benign verrucous lesions predominantly caused by HPV types 6 and 11 and affect the female external genitalia. Treatment aims to remove lesions, alleviate symptoms, reduce viral transmission, and prevent complications. Despite the availability of multiple therapeutic options, none is capable of eradicating HPV infection, and recurrence rates remain high, ranging from 5% to 20% [4], particularly among immunosuppressed individuals, including those living with HIV or using immunosuppressive drugs [2]. One of the main clinical concerns is the potential progression of these lesions to malignancy, given the established association between HPV infection and vulvar cancer [5]. Nevertheless, current evidence indicates that the overall contribution of HPV to invasive vulvar cancer may have been overestimated.

Therefore, the aim of this study is to evaluate clinical progression, treatment response, and recurrence in a group of Brazilian immunosuppressed patients with HPV-associated vulvar condylomas, compared with immunocompetent patients.

## Method

### Design and ethical issues

This retrospective cohort study was approved by the local Research Ethics Committee under number 6.566.826 and conducted in accordance with the Good Clinical Practice Guidelines and the Declaration of Helsinki. The study involved a review of patients’ medical records and covered a thirteen-year period (January 2008 to January 2021). All patients were attended by the same local Cervical Pathology Service at Hospital Erasto Gaertner, Curitiba, Paraná, Brazil.

### Participants

We included adult women diagnosed with vulvar condyloma (ICD-10 code B07), caused by HPV, who were clinically classified as immunosuppressed in the following situations: women living with HIV, with or without antiretroviral therapy; women with autoimmune diseases receiving immunosuppressive drugs and women undergoing cancer treatment with chemotherapy.

Pregnant women, patients with non-genital condylomas, patients who did not complete at least 12 months of follow-up, and those with incomplete medical records were excluded.

The comparison group comprised women diagnosed with vulvar condyloma who were clinically classified as immunocompetent, matched for age and comorbidities.

### Data collection

The variables collected and analyzed included age, cause of immunosuppression, CD4 + T-lymphocyte count in HIV-positive patients, number of condylomas, lesion extent, type of treatment for condyloma, need for multiple treatment sessions, treatment leading clinical remission and clinical outcome.

Clinical remission was defined as the absence of lesions at the end of 1-year of post-treatment follow-up. Recurrence was defined as the reappearance of lesions after appropriate treatment and documented clinical improvement during the 1-year clinical follow-up period. Disease progression was defined as the presence of a lesion with clinical features suggestive of high-grade intraepithelial neoplasia and histopathological confirmation of High-Grade Vulvar Intraepithelial Neoplasia (HSIL/VIN usual type) and/or invasive neoplasia.

HPV genotyping data could not be analyzed because the study was conducted within a public health service in a developing country that did not have this technology available during the study period.

Data regarding smoking habits in the medical records were highly incomplete (time of use, number of cigarettes per day, current vs. past habits), so the decision was made to not use this variable.

### Statistical analysis

Data were organized in frequency tables. Statistical analyses were performed using SPSS version 17.0. The Kolmogorov–Smirnov and Shapiro–Wilk tests were used to assess data normality. Continuous variables were expressed as mean ± standard deviation (SD) or median and interquartile range (IQR), and compared using the Mann–Whitney U test. Categorical variables were expressed as frequencies and percentages and compared using Fisher’s exact test or the chi-square test, as appropriate. P values < 0.05 were considered statistically significant.

## Results

Based on the ICD-10 code for viral condyloma, 1,120 patients treated at the service during the study period were initially identified. Of these, approximately two-fifths had non-genital condylomas and one-fifth were men with condylomas; these patients were excluded, leaving 448 potentially eligible women with genital condylomas. Patients who were pregnant, did not meet the predefined age criteria, or did not complete the recommended follow-up were subsequently excluded. After applying these eligibility criteria, 294 women were included in the final study sample.

The studied sample comprised 294 patients, with mean age was 37.8 ± 8.25 years (range: 18–86 years). Among them, 41/294 (13.9%) were classified as immunosuppressed and 253/294 (86.1%) as immunocompetent. Among the immunosuppressed group, 25/41 (61.0%) were HIV-positive, 6/41 (14.6%) were diagnosed with systemic lupus erythematosus (SLE), and 10/41 (24.4%) had a neoplasm. Follow-up time ranged from 12 to 144 months (median: 72 months).

Table 1 summarizes the clinical characteristics of the lesions and treatment modalities in both groups. While the number of lesions did not differ significantly between immunosuppressed and immunocompetent patients, lesion extent was significantly greater among immunosuppressed women. Regarding first-line treatment, trichloroacetic acid (TCA) was significantly more effective in immunocompetent patients. Likewise, the clinical remission rate after first line treatment was significantly higher among immunocompetent women (55% vs. 21.9%, *p* < 0.0001; OR = 4.5; IC95 = 1.9–9.3). Similarly, the complete remission rate at the end of follow-up was also statistically higher for the immunocompetent patients (75.9% vs. 48.8%, *p* < 0.0001, OR = 3.3; CI95%=1.6–6.5).

**Table 1:** Comparative clinical characteristics of condylomas in immunosuppressed versus immunocompetent patients groups

	Immuno-compromised *n* = 41	Immuno-competent *n* = 253	*p* value / OR / CI95%
Number of condylomasSingleMultiple	0 (0%) 41 (100%)	16 (6%) 237 (94%)	*p* = 0.14
*Lesion extension* 50% of vulve All vulve Vulve and perianal	7 (16%) 23 (57%) 11 (26%)	100 (40%) 73 (29%) 80 (31%)	*p* = 0.0005* OR = 3.2; CI95%=1.7–6.4
*First treatment drug* Tricloroacetic acid Imiquimod Ressection Combination	22 (55%) 13 (31%) 2 (5%) 4 (9.5%)	211 (83%) 23 (9%) 17 (7%) 2 (0.7%)	*p* < 0.0001**
*Clinical remission after first treatment /* Yes	9 (21.9%)	140 (55%)	*p* < 0.0001 OR: 4.5; CI95%=1.9–9.3
*Which treatment led to clinical remission?* Tricloroacetic acid Combination Imiquimod Ressection Exeresis None	10 (24%) 7 (17%) 3 (7%) 5 (12%) 0 (0%) 22 (53%)	144 (56%) 11 (4%) 19 (8%) 25 (10%) 3 (2%) 51 (20%)	*p* < 0.0001**
*Outcome* Clinical remission Persistent lesion	20 (48.8%) 21 (51.2%)	192 (75.9%) 61 (24.1%)	*p* < 0.0001 OR: 3.3; CI95%=1.6–6.5

* Risk to lesion in all vulve; ** Referent the use of Tricloroacetic acid

Among the immunosuppressed patients, 60% (*n* = 25) were seropositive. Of these, 72% (*n* = 18) had a CD4 + T-lymphocyte count ≥ 200, and 28% (*n* = 7) had a CD4 + T-lymphocyte count < 200. Among them 80% (*n* = 20) had a detectable viral load and 20% (*n* = 5) had an undetectable one. The overall recurrence rate among HIV patients was 24% (6/25), compared to 13.3% (36/269) among all HIV-negative patients (*p* = 0.11; OR: 2.9; 95% CI: 0.8–5.8).

In HIV-positive patients, when considering CD4 + T-lymphocyte count, 11.1% (*n* = 2) of those with CD4 ≥ 200 experienced recurrence, compared to 28.5% among those with CD4 < 200, although the difference was not statistically significant (*p* = 0.25). Similarly, patients with detectable viral load did not have a higher recurrence rate (20% in both groups).

Table 2 compares the two groups regarding lesion recurrence after treatment, disease progression, and patterns of clinical evolution, including the development of vulvar intraepithelial neoplasia or cancer. Immunosuppressed women exhibited a 2.3-fold higher likelihood of lesion recurrence compared with immunocompetent patients (*p* = 0.04), as well as a significantly increased risk of disease progression to invasive vulvar carcinoma (OR = 8.5; CI95%=2.4–29.3) and intraepithelial neoplasia (OR = 6.7; CI95% 1.8–24.0).

**Table 2:** Comparative clinical characteristics regarding condyloma recurrence and progression to intraepithelial or invasive disease in immunosuppressed versus immunocompetent groups

	Immuno-compromised *n* = 41	Immuno-competent*n* = 253	*p* value / OR/CI95%
Recurrence of lesion Yes	10 (24.4%)	30 (11.8%)	*p* = 0.04 OR: 2.3 (CI95% 1.0–5.2)
*Progression of the invasive disease?* Yes	6 (14.6%)	5 (2%)	*p* = 0.0014 OR: 8.5 (CI95% 2.4–29.3)
*Outcome* Intraepitelial Neoplasia Vulvar Cancer	5 (12.1%) 1 (2.3%)	5 (2%) 0 (0%)	*p* = 0.00067 OR: 6.7 (CI95% 1.8–24.0)

* Risk of intraepitelial neoplasia

Among immunosuppressed women, recurrence rates, varied according to the underlying condition, occurring in 24% of HIV-positive patients, 33% of those with SLE, and 10% of patients undergoing cancer treatment; however, these differences were not statistically significant.

## Discussion

This study provides insight into the clinical course and treatment response of HPV-related vulvar lesions in immunosuppressed Brazilian women compared with immunocompetent counterparts. The findings indicate that immunosuppressive conditions are associated with compromised immune function, which correlates with more extensive disease, reduced responsiveness to first-line therapies, and a higher risk of malignant progression. These results have important implications for clinical practice. Although vulvar condylomas are often managed with relatively straightforward and rapid treatment in immunocompetent patients, immunosuppressed women require closer monitoring, counseling regarding the increased likelihood of recurrence, and preparedness for prolonged or repeated therapeutic interventions. Furthermore, the elevated risk of progression underscores the need for vigilant surveillance and individualized management strategies in this high-risk population.

In our study, lesion extent differed markedly between groups, with more than half of the immunosuppressed patients presenting involvement of the entire vulva, compared with 29% among immunocompetent women. The clinical expression of HPV infection is influenced by a complex interaction between host-related factors—such as hormonal cyclic variations, the mucocutaneous microenvironment, local microbiota composition, and systemic immune competence—and viral characteristics, including immune evasion mechanisms [6]. This multifactorial interplay likely contributes to the greater extent and severity of HPV-related disease observed in immunosuppressed individuals [7].

Regarding therapeutic efficacy after the first intervention, outcomes were significantly poorer among immunosuppressed patients, with only 21% achieving complete remission following initial treatment, compared with 55% of immunocompetent women, and a higher need for multiple treatment sessions. Trichloroacetic acid (TCA) was the most frequently used therapy in both groups; however, clinical remission was achieved in only 24% of immunosuppressed patients treated with TCA, compared with 52% among immunocompetent patients. These findings are consistent with previous reports in the literature, which highlight the challenging nature of HPV-related lesions to treat, not only in immunosuppressed patients but also in the general population [2]. Imiquimod is a therapeutic option that could have been more widely used as part of the treatment strategy. In the studied sample, only a small proportion of patients reported using this medication. This limited use is likely attributable to the lack of availability of imiquimod within the Brazilian Unified Health System (SUS) and its higher cost compared with other therapeutic options [8]. 

In the studied sample, immunosuppressed women exhibited a markedly higher risk of progression to neoplasia, a finding of major clinical concern. This increased susceptibility may be partly explained by alterations in the tumor immune microenvironment in HPV-associated neoplasia, which can be broadly classified into active and exhausted immune phenotypes [9]. The active immune subtype is characterized by a robust interferon (IFN) signature and dense infiltration of immune effector cells, promoting effective antitumor responses, whereas the exhausted immune subtype displays immunosuppressive and pro-tumor features, including stromal activation, activation of the TGF-β signaling pathway, reduced CD8⁺ T-cell infiltration, and expression of wound-healing–related genes [9]. Accordingly, immunocompromised patients are more likely to exhibit impaired antitumor immune surveillance, increasing the risk of malignant progression and unfavorable outcomes [10]. In HPV-associated cervical and vulvar carcinogenesis, immune cell infiltration often coexists with marked immune evasion mechanisms driven by viral oncoproteins E5, E6, and E7, which promote T-cell exhaustion, upregulation of PD-L1, and suppression of antigen presentation through downregulation of MHC class I and II molecules [11]. These mechanisms are further amplified in immunosuppressed individuals, whose ability to regenerate functional effector immune cells is already compromised [12]. 

The most frequent neoplastic complication in the studied population was vulvar intraepithelial neoplasia, with an overall prevalence of 3.4%. This complication was significantly more common among immunosuppressed patients and carries a potential risk of progression to invasive carcinoma [2]. Vulvar diseases encompass a wide range of etiologies, including inflammatory dermatoses, pain syndromes, and premalignant conditions, often requiring specialized care and multidisciplinary collaboration. Accurate differentiation between benign findings and lesions with premalignant or malignant potential is essential to guide appropriate management and ensure high-quality, patient-centered care [13, 14]. 

Several guidelines acknowledge that immunosuppression promotes more extensive anogenital lesions, with higher rates of treatment failure and higher rates of recurrence. However, data do not support a therapeutic distinction for immunocompetent patients but suggest closer monitoring due to the increased risk of progression to invasion in immunocompromised patients [2, 14–16]. 

This study has several limitations inherent to its retrospective design, which makes it appropriate for hypothesis generation but limits causal inference. Smoking status and HPV genotype were not available for analysis, although both are established determinants of HPV persistence and progression to VIN and may have influenced the observed differences between groups. Other limitations include incomplete data capture and the inability to assess late recurrence, progression to other genital or anal neoplasms, and treatment-related complications. An additional limitation is the etiological heterogeneity of the immunosuppressed group. Although these conditions involve distinct mechanisms of immunosuppression that may differentially affect HPV persistence and treatment response, the small number of patients in each subgroup precluded adequately powered subgroup analyses. Larger multicenter studies are needed to investigate the potential impact of different causes of immunosuppression on clinical outcomes. Additionally, the study population predominantly comprised individuals of low socioeconomic status, which may have contributed to higher rates of loss to follow-up and limited access to therapeutic modalities considered more effective according to current medical literature. On the other hand, this study addresses a clinically relevant and underexplored topic, providing real-world data from a tertiary referral center over an extended period and the findings demonstrate a biologically plausible association between immunosuppression and increased recurrence and neoplastic progression. Additionally, this study contributes valuable data from Latin America, a region underrepresented in gynecologic oncology research. Given the limited financial resources available for research in developing countries, despite the substantial burden of these conditions, greater investment from high-income countries could facilitate well-designed, prospective controlled studies capable of addressing these important unanswered questions.

HPV vaccines have the potential to substantially reduce the incidence of HPV-related cancers; however, vaccination coverage varies worldwide and remains suboptimal in many settings. In Brazil, a free national HPV vaccination program was implemented in 2014 with a target coverage of 80%, yet effective uptake has remained below expectations. Limited knowledge about HPV infection and the benefits of vaccination contributes to vaccine hesitancy, indicating that improved information for women’s and children’s health policymakers is essential. Nevertheless, education alone may be insufficient to change health behaviors, highlighting the need for targeted awareness campaigns in healthcare settings, schools, and the media to overcome barriers to vaccination [17]. In addition, the use of condoms during sexual intercourse, regular gynecological follow-up, preventive screening, and discouragement of smoking are essential measures to reduce the population viral load, the risk of precursor lesions, and the incidence of gynecological cancer — thus promoting health and quality of life through education and prevention [18, 19]. 

In conclusion, in the studied sample, immunosuppressed women with vulvar condylomas present more extensive disease, poorer response to initial treatment, higher recurrence rates, and a markedly increased risk of progression to neoplasia. These findings underscore the need for closer surveillance, individualized treatment strategies, and targeted preventive measures in this high-risk population.

## Data Availability

The data presented in this study are available on request from the corresponding author.
